# Exertional Chest Pain and Dyspnea From Hyperviscosity Syndrome Related to Marginal Zone Lymphoma

**DOI:** 10.14740/wjon837w

**Published:** 2014-08-25

**Authors:** Prajwal Dhakal, Vijaya Raj Bhatt, Smrity Upadhyay, Nabin Khanal, Apar Kishor Ganti

**Affiliations:** a Department of Medicine, Institute of Medicine, Tribhuvan University, Kathmandu, Nepal; b Department of Internal Medicine, Division of Hematology-Oncology, University of Nebraska Medical Center, Omaha, NE, USA; c Department of Internal Medicine, Creighton University Medical Center, Omaha, NE, USA; d Department of Internal Medicine, Veteran’s Affairs Nebraska-Western Iowa Health Care System, Omaha, NE, USA

**Keywords:** Hyperviscosity syndrome, Marginal zone lymphoma, Chest pain, Dyspnea, Bendamustine, Rituximab

## Abstract

Hyperviscosity syndrome is usually seen in Waldenstrom macroglobulinemia with the serum viscosity > 4 cP. It commonly manifests as skin or mucosal bleeding, visual abnormalities and neurological symptoms. We describe a case of a 65-year-old man, who presented with chronic exertional chest pain and dyspnea without other manifestation and had hyperviscosity syndrome related to marginal zone lymphoma (MZL) even with a marginally elevated plasma viscosity. The symptoms resolved after initiation of chemotherapy. This case illustrates that MZL can cause hyperviscosity syndrome, which can manifest with cardiopulmonary symptoms without other clinical features even at a marginally elevated plasma viscosity. Atypical cases of hyperviscosity syndrome can, thus, present diagnostic challenges and require a high index of suspicion.

## Introduction

Exertional chest pain and dyspnea are frequently the presenting symptoms of ischemic heart disease. Additionally, several non-cardiac causes such as chronic obstructive pulmonary disease, pneumonia and anemia may also result in these symptoms. Many of these disorders are associated with specific clinical features or have specific findings on a routine evaluation such as a chest X-ray, hence they are relatively easy to diagnose. Although uncommon, hyperviscosity syndrome is an important cause of unexplained dyspnea and chest pain, especially in those with an underlying hematologic disorder [1, 2]. We report a case of exertional chest pain and dyspnea due to hyperviscosity syndrome, but without other clinical features of the syndrome. This case highlights the importance of a high index of suspicion for the diagnosis of a subtle hyperviscosity syndrome.

## Case Report

A 65-year-old man presented to a primary care clinic with exertional chest pain radiating to both arms for the last 2 years, progressively increasing over the last couple of months. The pain was relieved with rest and was associated with exertional dyspnea. The patient denied any weight loss, fevers, night sweats, headache, blurry vision or any neurologic symptoms. Physical examination demonstrated pallor and splenomegaly palpable 4 cm below the left costal margin. The remainder of the examination was unremarkable.

Laboratory tests revealed a white blood cell count of 2,500/µL, hemoglobin of 6.5 g/dL and platelet of 117,000/µL. A comprehensive metabolic panel was remarkable only for an elevated total protein level of 10.1 g/dL, whereas creatinine (1.0 mg/dL), calcium (9.5 mg/dL) and albumin (4.0 g/dL) levels were normal. Serial cardiac enzymes, electrocardiogram and echocardiogram were essentially normal. With blood transfusion, hemoglobin increased to 8.2 g/dL without any symptom improvement. Further tests demonstrated an elevated IgM (> 2,400 mg/dL), IgM kappa monoclonal protein of 3.33 g/dL and plasma viscosity of 3.1 cP. Bone marrow biopsy revealed atypical lymphoid cells with minimal cytoplasm and condensed chromatin, positive for CD 19 and CD20 but negative for CD3, CD5, CD 23 and cyclin D1. Cytogenetics was 46XY whereas fluorescence *in situ* hybridization showed concurrent deletions of 6q21 and 6q23 (10%) and three copies of 5q22 (47%). Positron emission tomography scan revealed splenomegaly (21 cm) with a maximum standardized uptake value of 3.4 and mildly enlarged diffuse hypermetabolic bilateral axillary, periportal, and iliac lymph nodes with a maximum standardized uptake value of 4.1.

With the diagnosis of marginal zone lymphoma (MZL), the patient was treated initially with a cycle of bendamustine. Rituximab was omitted in the first cycle to avoid paradoxical increase in serum IgM and viscosity. There was dramatic resolution of his symptoms after the first cycle. Repeat laboratory tests revealed IgM > 2,680 mg/dL, monoclonal protein of 2.0 g/dL and plasma viscosity of 2.3 cP. Thus, a retrospective diagnosis of hyperviscosity syndrome due to MZL was established. Subsequently, he received five cycles of bendamustine and rituximab with resolution of splenomegaly and improvement of blood counts. Ten months after the diagnosis, he continues to do well and works 30 h a week without any symptoms.

## Discussion

Hyperviscosity syndrome, seen in approximately 15% of patients with Waldenstrom macroglobulinemia, is the most serious and potentially fatal complication of macroglobulinemia. [1]. Waldenstrom macroglobulinemia, a clinicopathologic syndrome characterized by a proliferation of lymphocytes, is usually caused by lymphoplasmacytoid lymphoma or other small B-cell lymphoproliferative disorders such as chronic lymphocytic leukemia/small lymphocytic lymphoma. Even though hyperviscosity syndrome is more common in Waldenstrom macroglobulinemia [3], rarely extranodal marginal zone B-cell-mucosa-associated lymphoid tissue lymphoma, a subtype of MZL, has been associated with Waldenstrom macroglobulinemia-like syndrome [2, 4-7]. MZL is an indolent B-cell lymphoma arising from memory B cells in marginal zone of lymphoid tissue [8], which may lead to hyperviscosity syndrome as illustrated by this report.

Hyperviscosity syndrome commonly manifests as skin or mucosal bleeding, visual abnormalities, headache, vertigo, dizziness, nystagmus, deafness, ataxia and in severe cases confusion, dementia, stroke or coma [3, 9-14]. Pulmonary edema and congestive heart failure have also been reported [3, 9, 10, 12, 13]. However, as suggested by this report, uncommonly, hyperviscosity syndrome may manifest with exertional chest pain and dyspnea without other symptoms. Although this patient presented with significant anemia, blood transfusion led to an improvement in hemoglobin to 8.2 g/dL without any symptom improvement. However, symptoms improved with initiation of chemotherapy and reduction in plasma viscosity, thus confirming that the symptoms were related to hyperviscosity syndrome.

Elevated plasma viscosity along with characteristic symptoms can be diagnostic of hyperviscosity syndrome. Although it is usually seen with the plasma viscosity > 4 cP, the level at which patients become symptomatic is quite variable (“symptomatic threshold”) [3, 9, 12-14]. In this patient, symptoms manifested at a plasma viscosity of 3.1 cP. The presence of anemia with resulting marginal hypoxia may have contributed to the symptom manifestation at a marginally elevated plasma viscosity.

Laboratory evaluation of a suspected hyperviscosity syndrome should include serum protein electrophoresis and immunofixation, quantitative immunoglobulin levels and plasma viscosity measurements [2]. Additionally, a complete blood count, kidney and liver function tests, lactate dehydrogenage and disease-specific evaluation are performed. The immediate treatment relies on the physical removal of the IgM protein from the blood stream by plasmapheresis, while long-term management is aimed at the control of underlying disease [15]. There may be a transient increase in IgM levels after rituximab therapy [16-18]. The IgM flare phenomenon may be prevented by plasmapharesis or by avoiding rituximab for the first one or two chemotherapy cycles.

Rituximab, an IgG(1)-kappa monoclonal antibody that targets the CD20 antigen on the surface of malignant and normal B lymphocytes, has been utilized in the treatment of different types of MZL as a single agent or in combination with chemotherapy [19]. Bendamustine hydrochloride, a novel alkylating agent, has shown efficacy in treatment of indolent non-Hodgkin lymphoma and can be administered in combination with rituximab with good outcomes in MZL [20]. Splenectomy (in splenic MZL) and radiotherapy (in limited disease) may have limited roles in select patients [20]. Bendamustine alone during the first cycle followed by the combination of bendamustine and rituximab was utilized successfully in this patient.

### Conclusion

This case highlights several unique features: MZL as an etiology of hyperviscosity syndrome, manifestation with cardiopulmonary symptoms without other clinical features and manifestation of symptoms at a marginally elevated plasma viscosity. Atypical cases of hyperviscosity syndrome can, thus, present diagnostic challenges and require a high index of suspicion.

## Author Notes

The primary affiliation of Dr Ganti is Veteran’s Affairs Nebraska-Western Iowa Health Care System, Omaha, NE, USA.
